# Metabolism of the vacuolar pathogen *Legionella* and implications for virulence

**DOI:** 10.3389/fcimb.2014.00125

**Published:** 2014-09-09

**Authors:** Christian Manske, Hubert Hilbi

**Affiliations:** ^1^Max von Pettenkofer Institute, Faculty of Medicine, Ludwig-Maximilians UniversityMunich, Germany; ^2^Institute of Medical Microbiology, Faculty of Medicine, University of ZürichZürich, Switzerland

**Keywords:** amoeba, *Dictyostelium*, *Legionella*, macrophage, metabolism, nutrition, pathogen vacuole, type IV secretion

## Abstract

*Legionella pneumophila* is a ubiquitous environmental bacterium that thrives in fresh water habitats, either as planktonic form or as part of biofilms. The bacteria also grow intracellularly in free-living protozoa as well as in mammalian alveolar macrophages, thus triggering a potentially fatal pneumonia called “Legionnaires' disease.” To establish its intracellular niche termed the “*Legionella*-containing vacuole” (LCV), *L. pneumophila* employs a type IV secretion system and translocates ~300 different “effector” proteins into host cells. The pathogen switches between two distinct forms to grow in its extra- or intracellular niches: transmissive bacteria are virulent for phagocytes, and replicative bacteria multiply within their hosts. The switch between these forms is regulated by different metabolic cues that signal conditions favorable for replication or transmission, respectively, causing a tight link between metabolism and virulence of the bacteria. Amino acids represent the prime carbon and energy source of extra- or intracellularly growing *L. pneumophila*. Yet, the genome sequences of several *Legionella* spp. as well as transcriptome and proteome data and metabolism studies indicate that the bacteria possess broad catabolic capacities and also utilize carbohydrates such as glucose. Accordingly, *L. pneumophila* mutant strains lacking catabolic genes show intracellular growth defects, and thus, intracellular metabolism and virulence of the pathogen are intimately connected. In this review we will summarize recent findings on the extra- and intracellular metabolism of *L. pneumophila* using genetic, biochemical and cellular microbial approaches. Recent progress in this field sheds light on the complex interplay between metabolism, differentiation and virulence of the pathogen.

## Introduction

*Legionella pneumophila* is an environmental bacterium ubiquitously found in freshwater, where it is associated with biofilm communities (Lau and Ashbolt, 2009; Hilbi et al., 2011). Protozoan predators like amoebae are part of these communities and feed on bacteria residing within these biofilms. *L. pneumophila* has developed a way to survive and replicate within these free-living protozoa by forming a unique compartment called the *Legionella*-containing vacuole (LCV). The LCV is a pathogen vacuole, wherein *L. pneumophila* dodges lysosomal degradation by acquiring components of early and late endosomes, mitochondria, the endoplasmic reticulum and ribosomes (Isberg et al., 2009; Urwyler et al., 2009a; Hilbi and Haas, 2012). To establish this intracellular niche, the bacterial Icm/Dot type IV secretion system (T4SS) is essential, as it translocates around 300 different “effector” proteins into the host cell, many of which target central eukaryotic pathways like endocytic, secretory or retrograde vesicle trafficking by exploiting small GTPases, phosphoinositide lipids and other host factors (Hubber and Roy, 2010; Finsel et al., 2013; Haneburger and Hilbi, 2013; Rothmeier et al., 2013; Hoffmann et al., 2014a). Besides its natural protozoan hosts, *L. pneumophila* also replicates within human alveolar macrophages and epithelial cells, thus causing a severe pneumonia called Legionnaires' disease. Most processes involved in survival in protozoa or macrophages are very similar and appear to be evolutionarily conserved (Gao et al., 1997; Greub and Raoult, 2004; Hoffmann et al., 2014b). In addition to biofilm and protozoan niches, *Legionella* spp. are also naturally found in physically more challenging habitats, such as extremely acidic environments, antarctic freshwater lakes and water sources with temperatures over 60°C (Hilbi et al., 2011). Accordingly, these facultative intracellular bacteria are an example of a microorganism colonizing many different environmental niches.

To survive within its extra- and intracellular niches, *L. pneumophila* employs a biphasic life cycle, where it alternates between two different forms in response to environmental and metabolic stimuli (Molofsky and Swanson, 2004). In its transmissive form the pathogen is motile, resistant to environmental stress like nutrient starvation and infectious to host cells. In its replicative form the bacteria lack these traits but are able to replicate intracellularly (Rowbotham, 1986; Brüggemann et al., 2006). Further manifestations of *L. pneumophila* differentiation include a mature intracellular form (MIF) that develops late during infection (Garduno et al., 2002). MIFs are motile, metabolically inert, highly infectious and loaded with cytoplasmic inclusions of poly-3-hydroxybutyrate. Moreover, under harsh conditions, *L. pneumophila* appears to adopt a viable but non-culturable (VBNC) state (Steinert et al., 1997; Garcia et al., 2007; Al-Bana et al., 2014). To ensure bacterial survival in different environments, the biphasic life cycle of *L. pneumophila* is strictly regulated. Consequently, *L. pneumophila* employs a multitude of regulatory systems devoted to the control of gene expression, including transcriptional regulators and two-component systems (Molofsky and Swanson, 2004).

**Figure 1:**
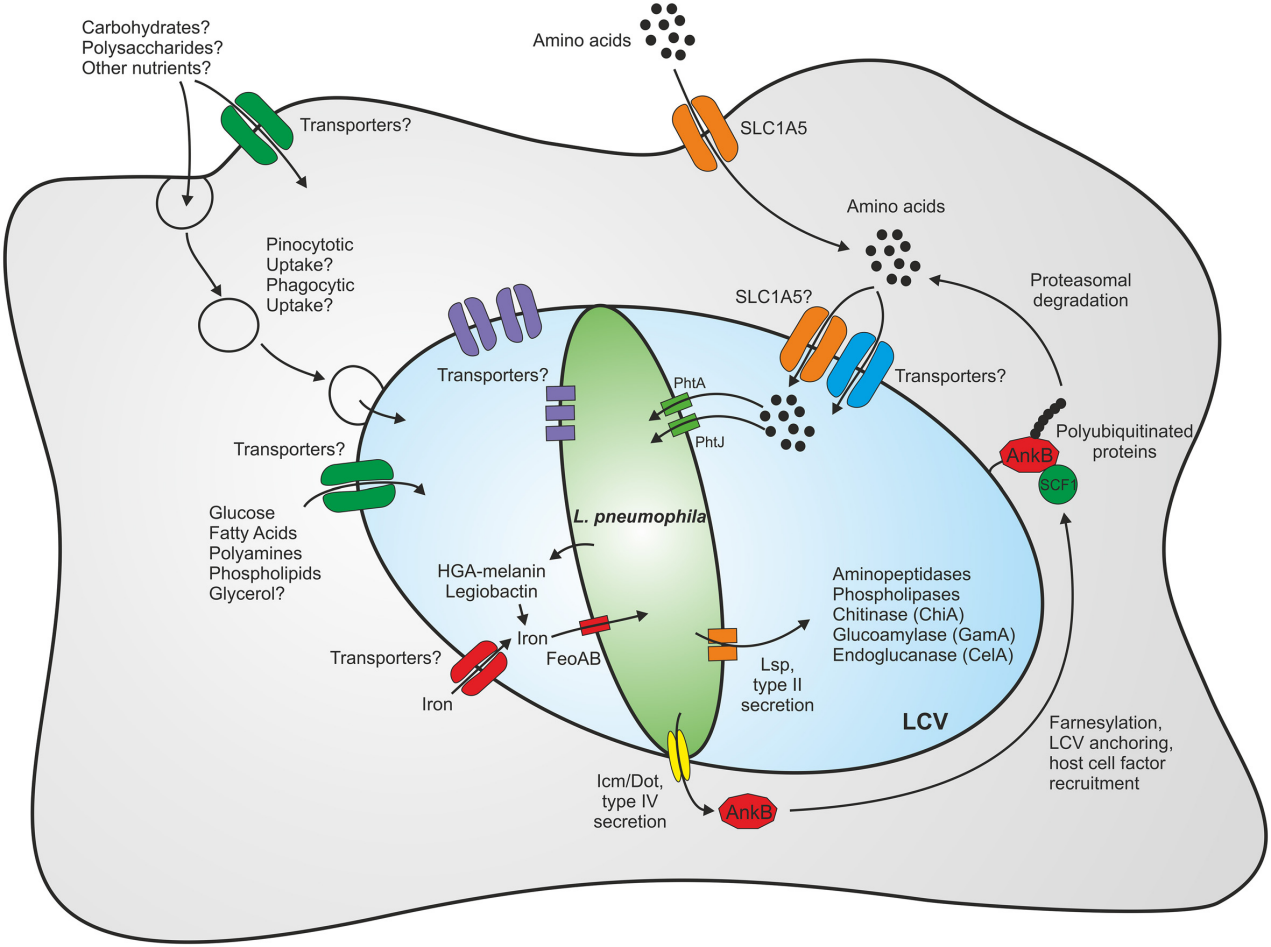
**Compartmentalization of the metabolism of *L. pneumophila***. Within eukaryotic host cells *L. pneumophila* forms a membrane-bound replication-permissive compartment, the *Legionella*-containing vacuole. The bacteria gain access to nutrients through intrinsic membrane transporters localizing to the plasma membrane or the pathogen vacuole membrane, respectively, or through fusion with host vesicles and compartments such as endosomes/macropinosomes or the endoplasmic reticulum. Amino acids represent the main carbon and energy source of *L. pneumophila*, yet carbohydrates and complex polysaccharides are also catabolized. For details see text.

As *L. pneumophila* survives in various environmental niches, it is likely that the bacterium exploits numerous different carbon and energy sources. Furthermore, the intracellular milieu might represent a richly set table for pathogens, as eukaryotic host cells contain many different nutrients, which are potentially accessible to intracellular pathogens (Eisenreich et al., 2010; Rohmer et al., 2011; Abu Kwaik and Bumann, 2013). An intriguing aspect of intracellular metabolism is its compartmentalization into processes that occur within the host cytoplasm, the LCV lumen or the bacteria (Figure 1).

Early metabolic studies suggested that amino acids are the major if not only source of carbon and energy for *L. pneumophila* (Pine et al., 1979; Tesh and Miller, 1981; Tesh et al., 1983). However, the subsequent availability of genome sequences, transcriptome, proteome and metabolism data indicated that *L. pneumophila* possess much broader metabolic capacities (Cazalet et al., 2004; Chien et al., 2004; Urwyler et al., 2009b; Eylert et al., 2010; Faucher et al., 2011; Hoffmann et al., 2014a; Schunder et al., 2014). In this review we will summarize the metabolic capacities of *L. pneumophila* regarding amino acid and carbohydrate degradation. Moreover, we will highlight further nutrient requirements of the bacteria and assess the regulation of their life cycle by metabolites.

## Amino acid metabolism

Initial studies of the nutrient requirements of *L. pneumophila* in chemically defined minimal media showed a preference for amino acids as main source of carbon and energy (Pine et al., 1979; Ristroph et al., 1981; Tesh and Miller, 1981; Tesh et al., 1983). A preference for amino acid utilization is also illustrated in the genome sequence of *L. pneumophila*, where around 12 classes of ATP binding cassette transporters, amino acid permeases and proteases can be found (Cazalet et al., 2004; Chien et al., 2004). Furthermore, genes involved in synthesis and transport of amino acids are highly induced during growth inside macrophages (Faucher et al., 2011). *L. pneumophila* employs transport systems to take up and utilize amino acids (Sauer et al., 2005), but also exploits host cell transporters (Wieland et al., 2005) and host proteolytic processes (Price et al., 2011).

*L. pneumophila* is an obligate aerobe organism and auxotroph for several amino acids including cysteine, arginine, isoleucine, leucine, threonine, valine, and methionine. The observed auxotrophy corresponds to the notion that cysteine biosynthetic genes and other anabolic genes are absent in the genomes of *L. pneumophila* (Cazalet et al., 2004; Chien et al., 2004; Glöckner et al., 2007; D'Auria et al., 2010; Schroeder et al., 2010) and *L. longbeachae* (Cazalet et al., 2010; Kozak et al., 2010). Compared to chemically defined media, the complex ACES-buffered yeast extract (AYE) broth routinely used to grow *L. pneumophila* contains several additional amino acids: alanine, asparagine, glutamine and glycine. The common solid growth medium for *Legionella* species is buffered charcoal-yeast extract (BCYE) agar, supplemented with L-cysteine and ferric pyrophosphate. *L. pneumophila* growth depends on excess cysteine in the medium (Feeley et al., 1979; George et al., 1980). Yet, the amount of cysteine added to the BCYE medium is much higher than what is required to support growth. The major part of cysteine in the *Legionella* growth medium is rapidly oxidized to cystine and becomes unavailable to the bacteria, as *L. pneumophila* is not able to utilize this compound (Ewann and Hoffman, 2006). The remaining concentration of cysteine is around 0.5 mM, which is enough to support *Legionella* growth. Furthermore, using radio-labeled cysteine and mutant strains, it was found that cysteine is not only imported by specific transporters but also consumed during *L. pneumophila* growth (Ewann and Hoffman, 2006).

*L. pneumophila* is also auxotroph for arginine, as the bacteria lack enzymes that allow synthesis of arginine from glutamate. However, the bacteria produce arginine in chemically defined medium supplemented with ornithine or citrulline, which are precursors of arginine emerging in the later steps of the synthesis from glutamate (Tesh and Miller, 1983; Hovel-Miner et al., 2010). Furthermore, *L. pneumophila* mutants lacking the arginine repressor ArgR fail to replicate within host cells. ArgR might sense the availability of arginine within the host. This leads to the expression of genes (many of them not involved in arginine metabolism), which are required for intracellular growth (Hovel-Miner et al., 2010).

The identification of the phagosomal transporter A (PhtA) revealed a major role of threonine not only for replication but also for differentiation of *L. pneumophila* (Sauer et al., 2005) (Figure 1). A mutant strain lacking *phtA* does not grow in a chemically defined medium, but is rescued by excess tryptone or dipeptides containing threonine, indicating that PhtA is not the only threonine uptake system. Intriguingly, *phtA* mutant bacteria are defective for intracellular replication in macrophages due to their inability to differentiate from the transmissive to the replicative state. Analogously, PhtJ was identified as a valine transporter also required for differentiation and replication within macrophages (Sauer et al., 2005; Chen et al., 2008). These findings highlight the role of the Pht transporters as means for *L. pneumophila* to scavenge amino acids from host cells.

Further evidence for the importance of the Pht transporter family for nutrient acquisition of *L. pneumophila* was obtained by investigating the *phtC-phtD* locus (Chen et al., 2008). The *phtC* and *phtD* genes are paralogs in an operon containing genes involved in nucleotide metabolism. The transporter genes are required for successful replication within macrophages and survival of thymidine deprivation. Expression of *phtC* and *phtD* in *E. coli* bestowed pyrimidine transport activity upon strains lacking all known nucleoside transporters, identifying PhtC and PhtD as thymidine transporters (Fonseca et al., 2014).

To take up and utilize amino acids, *L. pneumophila* does not only produce many own systems, but also exploits host metabolic functions (Figure 1). The eukaryotic neutral amino acid transporter SLC1A5 was found to be upregulated in *L. pneumophila*-infected cells, and blocking the transporter with the competitive inhibitor BCH (2-amino-2-bornonane-carboxylic acid) or depletion by RNA interference impaired intracellular growth of *L. pneumophila* (Wieland et al., 2005). This study demonstrated the requirement of a single host cell transporter for intracellular replication and also indicated that SLC1A5 may be recruited to the LCV, thus enabling *L. pneumophila* to import amino acids from the cytoplasm into the LCV lumen. Other host-cell transporters might be utilized in a similar manner. Notably, similar to *L. pneumophila*, *Francisella tularensis* modulates the expression of SLC1A5 upon infection of THP-1 human monocytes and is also impaired for intracellular replication when this transporter is downregulated (Barel et al., 2012).

*L. pneumophila* uses the Icm/Dot T4SS to translocate effector proteins across the LCV membrane to interfere with central host cell processes (Figure 1). The Icm/Dot substrate AnkB subverts amino acid metabolism and protein degradation by hijacking the host cell ubiquitination machinery and the proteasome to create nutrients for bacterial growth (Al-Khodor et al., 2010). AnkB harbors several eukaryotic domains: an F-box domain that allows interaction with the host SCF1 ubiquitin ligase complex, two ANK domains, which mediate protein-protein interactions in eukaryotes and a CaaX motif that is modified by farnesylation (Price et al., 2009, 2010a,b; Ensminger and Isberg, 2010; Ivanov et al., 2010; Lomma et al., 2010). Farnesylation of AnkB leads to localization of the effector to the LCV membrane, and intracellular replication of *L. pneumophila* fails when farnesylation is blocked. Anchoring of the effector to the LCV membrane recruits polyubiquitinated host cell proteins, which are degraded by the host proteasome generating a pool of amino acids utilized for intracellular bacterial replication (Price et al., 2011).

Isotopolog profiling is a powerful approach to study metabolic pathways. The method is based on the incorporation of carbon isotopes from stable isotope-labeled precursors such as [U-^13^C_3_]serine or [U-^13^C_6_]glucose. To elucidate the metabolic pathways and fluxes used, key metabolites such as protein-derived amino acids or storage compounds are then analyzed for the presence of labeled carbon atoms (Zamboni et al., 2009). Metabolomic flux analysis and isotopolog profiling have recently provided detailed insights into the metabolism of the pathogenic bacteria *Listeria monocytogenes* (Gillmaier et al., 2012) or *Streptococcus pneumonia* (Hartel et al., 2012), and the metabolic responses of infected host cells to the pathogens have also been investigated (Eisenreich et al., 2013).

Upon growth of *L. pneumophila* in AYE medium supplemented with [U-^13^C_3_]serine, incorporation of the ^13^C-label indicated that the amino acid was not only used for protein biosynthesis but also to synthesize other amino acids and poly-3-hydroxybutyrate (Eylert et al., 2010). Thus, in agreement with earlier studies (Pine et al., 1979; George et al., 1980; Ristroph et al., 1981) serine can serve as a major carbon source during growth of *L. pneumophila* in broth. Yet, no ^13^C-label was detected in isoleucine, leucine, phenylalanine, tyrosine, histidine, proline or valine, confirming the auxotrophy of *L. pneumophila* regarding these amino acids (Eylert et al., 2010). Finally, isotopolog profiling also revealed that *L. pneumophila* growing intracellularly in *Acanthamoeba castellanii* previously fed with [U-^13^C_6_]glucose utilizes amoebae-derived amino acids (e.g., phenylalanine, tyrosine) for protein biosynthesis (Schunder et al., 2014).

## Carbohydrate and polysaccharide metabolism

While amino acids seem to represent the preferred carbon source of *L. pneumophila*, the bacteria can also metabolize carbohydrates, other small organic compounds and complex nutrients (Figure 1). Early studies using ^14^C-radio-labeled substrates indicated that glucose, α-ketoglutarate, pyruvate, glycerol and acetate are metabolized by *L. pneumophila*, yet only some of these compounds stimulated extracellular bacterial growth under the conditions used (Pine et al., 1979; Weiss et al., 1980; Tesh et al., 1983). Moreover, during infection of macrophages *L. pneumophila* genes required for glycerol catabolism—namely *lpg1414* and *glpD*—were highly upregulated compared to growth in rich broth (Faucher et al., 2011). Therefore, glycerol likely plays a role during intracellular growth of *L. pneumophila*, similar to other intracellular bacteria such as *L. monocytogenes* (Eylert et al., 2008; Joseph et al., 2008) and *Salmonella enterica* (Steeb et al., 2013).

Glucose was not found to stimulate growth of *Legionella* spp.; however, the genomes of *L. pneumophila* (Cazalet et al., 2004; Chien et al., 2004; Glöckner et al., 2007; D'Auria et al., 2010; Schroeder et al., 2010) as well as *L. longbeachae* (Cazalet et al., 2010; Kozak et al., 2010) encode complete pathways required for metabolism of carbohydrates, including the Emden-Meyerhof-Parnas (EMP) pathway, the Entner-Doudoroff (ED) pathway, as well as an incomplete pentose phosphate (PP) pathway. In support of the notion that carbohydrate metabolism is crucial during infection, genes associated with the ED pathway, as well as a glucokinase and a glucoamylase, were upregulated upon intracellular growth of *L. pneumophila* in *A. castellanii* (Brüggemann et al., 2006). Another intracellular pathogen that depends on sugar assimilation via the ED pathway during intracellular growth is *S. enterica*. Yet, in this case the parallel exploitation of several different host nutrients enhances bacterial virulence (Steeb et al., 2013).

Using isotopolog profiling, it was recently shown that *L. pneumophila* indeed catabolizes glucose via the ED pathway (Eylert et al., 2010). Upon growth in a chemically defined medium containing [U-^13^C_6_]glucose, followed by analysis of the isotopolog pattern by mass spectrometry and NMR spectroscopy, the ^13^C-label was recovered with high efficiency in alanine and also in poly-3-hydroxybutyrate. In contrast, an *L. pneumophila* mutant lacking the glucose-6-phosphate dehydrogenase gene (*zwf*), the first gene of an operon comprising the genes of the ED pathway (*zwf*-*pgl*-*edd*-*glk*-*eda*-*ywtG*), did not incorporate label from glucose and was outcompeted by the wild-type strain in co-infection experiments using *A. castellanii* (Eylert et al., 2010). In line with these observations, *L. pneumophila* lacking other components of the ED pathway, either glucokinase (*glk*), phosphogluconate dehydratase (*edd*), 2-keto-3-deoxy-phosphogluconate aldolase (*eda*) or the putative sugar transporter (*ywtG*), was no longer able to metabolize glucose and was defective for growth in *Acanthamoeba culbertsoni* or mammalian cells (Harada et al., 2010). Together, these findings strongly support the notion that the ED pathway is essential for glucose metabolism and intracellular growth of *L. pneumophila*. The results also implicate that under the conditions prevailing within LCVs in host cells *L. pneumophila* does not solely grow on amino acids as carbon and energy sources, but rather, carbohydrates are also utilized (at least as co-metabolites). Yet, the relative contribution of amino acids and carbohydrates to intracellular growth is difficult to assess, and many carbohydrates do not support extracellular growth as sole source of carbon and energy.

The transporters promoting the uptake of sugars have not been studied in molecular detail at present. The gene *ywtG* (lpg0421) is conserved among *L. pneumophila* and *L. longbeachae*, and annotated as a putative D-xylose (galactose, arabinose)-proton symporter (Cazalet et al., 2004, 2010). However, arabinose appears to be barely taken up by *L. pneumophila* (excluding genetic approaches based on the arabinose promoter, P*_bad_*). Moreover, glucose-1-phosphate is metabolized much faster than glucose-6-phosphate or glucose, suggesting that the former compound is transported efficiently into the cells (Weiss et al., 1980).

In addition to simple carbohydrates and small organic compounds, polymeric compounds also likely serve as carbon sources for *L. pneumophila*. The exogenous supply of polyamines during infection moderately favored intracellular replication of *L. pneumophila* (Nasrallah et al., 2011). Moreover, similar to other bacteria (Khosravi-Darani et al., 2013), *L. pneumophila* might use the intracellular “energy reserve” poly-3-hydroxybutyrate as an endogenous source of carbon and energy, which is synthesized via pyruvate and acetyl-coenzyme A (James et al., 1999; Eylert et al., 2010). Further support for the notion that *Legionella* spp. degrade complex polysaccharides stems from the genome sequences. *L. longbeachae* harbors a number of genes likely involved in cellulose degradation (Cazalet et al., 2010), and *L. pneumophila* contains genes putatively involved in the degradation of cellulose, chitin, starch and glycogen (Cazalet et al., 2004).

The Lsp type II secretion system (T2SS) is essential for intracellular growth of *L. pneumophila* in amoebae and macrophages (Hales and Shuman, 1999a; Liles et al., 1999) (Figure 1). Proteome studies on the type II “secretome” of *L. pneumophila* revealed that the bacteria secrete a chitinase (ChiA), as well as an endoglucanase, which metabolizes carboxymethyl cellulose (Debroy et al., 2006). An endoglucanase (CelA) was indeed found to degrade cellulose (Pearce and Cianciotto, 2009), and a eukaryotic-like glycoamylase (GamA) degraded carboxymethyl cellulose, glycogen and starch (Herrmann et al., 2010). Yet, neither CelA nor GamA was required for growth of *L. pneumophila* in amoebae. In summary, insights from genomics, transcriptomics, metabolomics, as well as biochemical experiments indicate that *L. pneumophila* utilizes simple and also complex carbohydrates as important sources of carbon and energy during extra- and intracellular growth.

## Micronutrient requirements

Iron is essential for growth of most if not all bacteria, as it is a co-factor for many enzymes of the central metabolism as part of prosthetic groups like heme or iron-sulfur clusters (Ratledge and Dover, 2000). Moreover, the availability of iron is especially important for pathogens, as iron limitation plays an important role in host defense against infections. For *L. pneumophila* iron represents an essential nutrient and has to be supplemented in high concentrations to growth media (Reeves et al., 1981; Ewann and Hoffman, 2006). The major iron-containing protein of *L. pneumophila* is aconitase of the tricarboxylic acid cycle (Mengaud and Horwitz, 1993). *L. pneumophila* grown under iron-limited conditions showed reduced virulence and was impaired for survival in host cells (James et al., 1995). Furthermore, host cells treated with iron chelators did not support growth of *L. pneumophila*, presumably due to iron limitation, as the addition of iron as iron-transferrin or ferric iron-nitrilotriacetate reversed growth inhibition (Gebran et al., 1994; Byrd and Horwitz, 2000; Viswanathan et al., 2000). Notably, patients with iron overload or smokers are at increased risk for Legionnaires' disease, probably because their lungs contain increased levels of iron (Fields et al., 2002; Vikram and Bia, 2002).

Iron exists in equilibrium between a ferrous (Fe^2+^) and a ferric (Fe^3+^) form, depending mostly on the pH and availability of oxygen (Williams, 2012). In *L. pneumophila*, many systems are devoted to iron metabolism and involved in iron reduction, complexation and transport (Figure 1). Iron reductase enzymes may promote iron assimilation in the periplasm and cytoplasm (Johnson et al., 1991; Poch and Johnson, 1993). Iron reduction is also catalyzed by the secreted compound homogentisic acid (HGA) and its polymerized derivative HGA-melanin (Chatfield and Cianciotto, 2007; Zheng et al., 2013). HGA is a product of the phenylalanine and tyrosine catabolism of *L. pneumophila* and was identified as the brown pigment secreted by *L. pneumophila*, which is produced from oxidative polymerization of HGA to HGA-melanin (Steinert et al., 2001). HGA and HGA-melanin stimulate growth of *L. pneumophila* under iron-limiting conditions, enhance the uptake of iron and can release ferrous iron from transferrin and ferritin, two major protein iron chelators of mammalian cells (Zheng et al., 2013).

*L. pneumophila* chelates and transports iron with the secreted high-affinity iron siderophore legiobactin (Liles et al., 2000; Starkenburg et al., 2004). The gene *lbtA*, which has homology with siderophore synthetases, and *lbtB* that encodes a homolog of a multidrug efflux pump, were identified as key players in the synthesis of legiobactin (Allard et al., 2006). Moreover, an *L. pneumophila lbtA* mutant strain showed reduced ability to infect lungs of A/J mice, demonstrating the importance of legiobactin *in vivo* (Allard et al., 2009). Iron is also transported in *L. pneumophila* via the *FeoAB* system (Robey and Cianciotto, 2002). The *L. pneumophila feoAB* operon bears homology to the *E. coli* system, a well-characterized ATP-driven ferrous iron transporter. An *L. pneumophila feoB* mutant was outcompeted by wild-type bacteria during infection of A/J mice, highlighting an important role of the transporter *in vivo*.

Finally, in addition to iron, the extracellular growth of *L. pneumophila* is also stimulated by calcium, magnesium and zinc. Calcium and magnesium might also play a role in biofilm formation, as the two metal ions enhance the adherence of *Legionella* to surfaces (Reeves et al., 1981; Koubar et al., 2013).

## Metabolic regulation of differentiation and virulence

**Figure 2:**
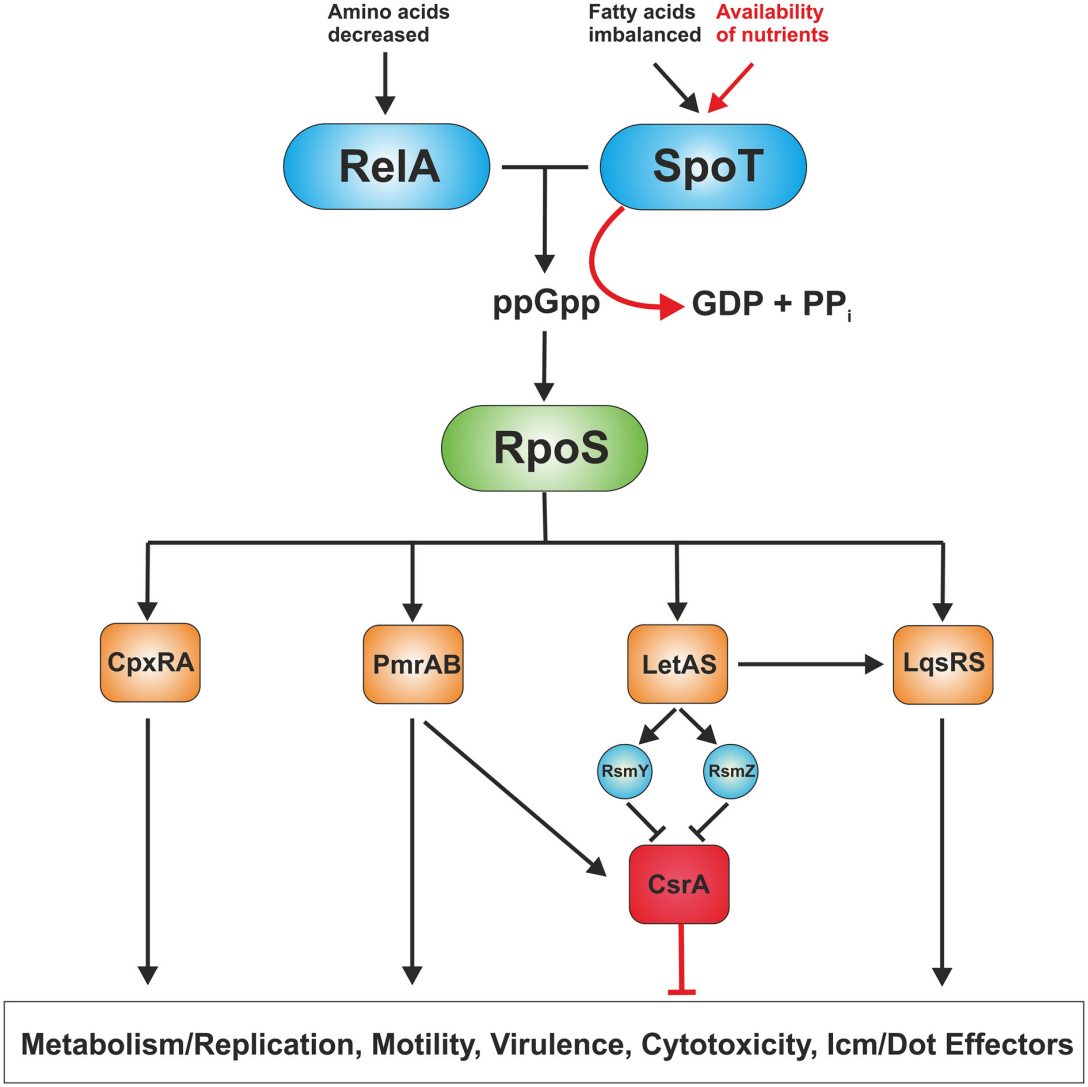
**Regulation of replicative and transmissive traits of *L. pneumophila***. *L. pneumophila* senses its metabolic state by means of the two ppGpp synthases RelA and SpoT. RelA detects amino acid starvation, whereas SpoT monitors disturbances in fatty acid synthesis. When nutrients become limiting, the “alarmone” ppGpp acumulates in the bacteria leading to production of the alternative sigma factor RpoS. In turn, RpoS regulates the two component or quorum sensing systems CpxRA, PmrAB, LetAB, and LqsRS, which control metabolism/replication, motility as well as virulence traits, and hence, govern the transition from the replicative to the transmissive form. The RNA-binding global regulator CrsA controls the biphasic switch as an antagonist of the two component and quorum sensing systems.

The biphasic life cycle of *L. pneumophila* is regulated by a variety of environmental and metabolic stimuli (Molofsky and Swanson, 2004). As long as nutrients are not limiting, the post-transcriptional regulator CsrA suppresses transmission traits and promotes replication (Molofsky and Swanson, 2003). Given the importance of amino acids as carbon source for *L. pneumophila*, it is not surprising that these compounds are also main regulatory factors of the phenotypic switch (Byrne and Swanson, 1998; Sauer et al., 2005). Amino acid starvation or otherwise nutrient-limiting conditions trigger the shift from the replicative/non-motile to the virulent/motile form of *L. pneumophila*, which is mediated through the second messenger guanosine 3′,5′-bispyrophosphate (ppGpp) (Hammer and Swanson, 1999; Dalebroux et al., 2010) (Figure 2). The “alarmone” ppGpp is synthesized by the synthase RelA as part of the “stringent response” that senses the accumulation of uncharged tRNAs at the ribosome. A second stringent response enzyme called SpoT also synthesizes ppGpp (Dalebroux et al., 2009). However, rather than sensing amino acid shortage, SpoT monitors fatty acid biosynthesis by interacting with the acyl-carrier protein ACP (Edwards et al., 2009). In addition, SpoT hydrolyzes ppGpp during exponential growth to ensure that transmissive traits are not expressed during replication.

The alternative sigma factor RpoS (σ^38^/σ^S^) represents the pivotal transcriptional regulator of the *L. pneumophila* life cycle (Hales and Shuman, 1999b; Bachman and Swanson, 2001; Zusman et al., 2002). An *L. pneumophila rpoS* mutant is not affected regarding extracellular growth in broth and retains significant stress resistance, but is not able to replicate in amoebae. This severe defect in intracellular replication is not due to impaired Icm/Dot function or *icm*/*dot* gene expression, but because of major transcriptional changes affecting basic cellular processes and other central regulatory networks (Hovel-Miner et al., 2009). The transcription of more than 70 genes required for central metabolism, 40 of these associated with amino acid metabolism, was negatively regulated in the *rpoS* mutant. Furthermore, small regulatory RNAs (*rsmY* and *rsmZ*) (Rasis and Segal, 2009; Sahr et al., 2009), two component systems (CpxRA, PmrAB), the transcriptional regulator ArgR and the quorum sensing response regulator LqsR (Tiaden et al., 2007) are regulated by RpoS (Bachman and Swanson, 2004; Hovel-Miner et al., 2009) (Figure 2).

At least three two component systems and one quorum sensing system influence the virulence of *L. pneumophila:* CpxRA (Gal-Mor and Segal, 2003a; Altman and Segal, 2008), PmrAB (Zusman et al., 2007; Al-Khodor et al., 2009; Rasis and Segal, 2009), LetAS (GacAS) (Hammer et al., 2002; Gal-Mor and Segal, 2003b; Lynch et al., 2003) and the *Legionella* quorum sensing (*lqs*) gene cluster (Tiaden et al., 2010) (Figure 2). The *lqs* system of *L. pneumophila* comprises the autoinducer synthase LqsA, the sensor kinases LqsS and LqsT (Kessler et al., 2013), and the response regulator LqsR (Tiaden et al., 2007). LqsA produces the compound LAI-1 (*Legionella* autoinducer-1, 3-hydroxypentadecane-4-one) (Spirig et al., 2008), which presumably binds to the cognate sensor kinases. The kinase-mediated phosphorylation signal converges on LqsR (Schell et al., 2014), which among many other processes also controls the switch from the stationary phase to the replicative phase (Tiaden et al., 2007). Lqs-regulated processes include pathogen-phagocyte interactions, production of extracellular filaments, natural competence for DNA uptake and the expression of a 133 kb genomic “fitness island” (Tiaden and Hilbi, 2012). Furthermore, transcriptome analysis of *L. pneumophila* strains lacking *lqsR*, *lqsS* or *lqsT* or the entire *lqs* cluster indicates that the Lqs system also regulates a number of metabolic pathways (Tiaden et al., 2007, 2008; Kessler et al., 2013).

## Conclusions and perspectives

The amoebae-resistant bacterium *L. pneumophila* colonizes a variety of extra- and intracellular niches in the environment. Upon reaching the human lung, *L. pneumophila* grows in mammalian macrophages and possibly also in epithelial cells. Accordingly, the bacteria are equipped to utilize a broad range of compounds as carbon and energy sources. In addition to amino acids, which initially have been regarded as the main if not sole nutrients, carbohydrates have recently been shown to be catabolized by extra- and intracellularly growing *L. pneumophila*. Novel technological approaches such as isotopolog profiling allow analyzing metabolic fluxes with unprecedented resolution and sensitivity. Transcriptome and genome studies indicate that a number of other compounds, including complex polysaccharides, are also metabolized by *L. pneumophila*. Further studies will unravel the manifold and robust metabolic pathways that the bacteria employ to thrive in diverse environmental niches. Importantly, future investigations will also shed light on the intricate relationship between the physiology and pathogenesis of *L. pneumophila*, and thus might contribute to control Legionnaires' disease.

### Conflict of interest statement

The authors declare that the research was conducted in the absence of any commercial or financial relationships that could be construed as a potential conflict of interest.

## References

[B1] Abu Kwaik Y. Bumann D. (2013). Microbial quest for food *in vivo*: ‘nutritional virulence’ as an emerging paradigm. Cell. Microbiol. 15, 882–890 10.1111/cmi.12138 23490329

[B2] Al-Bana B. H. Haddad M. T. Garduno R. A. (2014). Stationary phase and mature infectious forms of *Legionella pneumophila* produce distinct viable but non-culturable cells. Environ. Microbiol. 16, 382–395 10.1111/1462-2920.12219 23968544

[B3] Al-Khodor S. Kalachikov S. Morozova I. Price C. T. Abu Kwaik Y. (2009). The PmrA/PmrB two-component system of *Legionella pneumophila* is a global regulator required for intracellular replication within macrophages and protozoa. Infect. Immun. 77, 374–386 10.1128/IAI.01081-08 18936184 PMC2612241

[B4] Al-Khodor S. Price C. T. Kalia A. Abu Kwaik Y. (2010). Functional diversity of ankyrin repeats in microbial proteins. Trends Microbiol. 18, 132–139 10.1016/j.tim.2009.11.004 19962898 PMC2834824

[B5] Allard K. A. Dao J. Sanjeevaiah P. McCoy-Simandle K. Chatfield C. H. Crumrine D. S. (2009). Purification of legiobactin and importance of this siderophore in lung infection by *Legionella pneumophila*. Infect. Immun. 77, 2887–2895 10.1128/IAI.00087-09 19398549 PMC2708552

[B6] Allard K. A. Viswanathan V. K. Cianciotto N. P. (2006). *lbtA* and *lbtB* are required for production of the *Legionella pneumophila* siderophore legiobactin. J. Bacteriol. 188, 1351–1363 10.1128/JB.188.4.1351-1363.2006 16452417 PMC1367248

[B7] Altman E. Segal G. (2008). The response regulator CpxR directly regulates expression of several *Legionella pneumophila icm/dot* components as well as new translocated substrates. J. Bacteriol. 190, 1985–1996 10.1128/JB.01493-07 18192394 PMC2258895

[B8] Bachman M. A. Swanson M. S. (2001). RpoS co-operates with other factors to induce *Legionella pneumophila* virulence in the stationary phase. Mol. Microbiol. 40, 1201–1214 10.1046/j.1365-2958.2001.02465.x 11401723

[B9] Bachman M. A. Swanson M. S. (2004). Genetic evidence that *Legionella pneumophila* RpoS modulates expression of the transmission phenotype in both the exponential phase and the stationary phase. Infect. Immun. 72, 2468–2476 10.1128/IAI.72.5.2468-2476.2004 15102753 PMC387865

[B10] Barel M. Meibom K. Dubail I. Botella J. Charbit A. (2012). *Francisella tularensis* regulates the expression of the amino acid transporter SLC1A5 in infected THP-1 human monocytes. Cell. Microbiol. 14, 1769–1783 10.1111/j.1462-5822.2012.01837.x 22804921

[B11] Brüggemann H. Hagman A. Jules M. Sismeiro O. Dillies M. A. Gouyette C. (2006). Virulence strategies for infecting phagocytes deduced from the *in vivo* transcriptional program of *Legionella pneumophila*. Cell. Microbiol. 8, 1228–1240 10.1111/j.1462-5822.2006.00703.x 16882028

[B12] Byrd T. F. Horwitz M. A. (2000). Aberrantly low transferrin receptor expression on human monocytes is associated with nonpermissiveness for *Legionella pneumophila* growth. J. Infect. Dis. 181, 1394–1400 10.1086/315390 10762570

[B13] Byrne B. Swanson M. S. (1998). Expression of *Legionella pneumophila* virulence traits in response to growth conditions. Infect. Immun. 66, 3029–3034 9632562 10.1128/iai.66.7.3029-3034.1998 PMC108309

[B14] Cazalet C. Gomez-Valero L. Rusniok C. Lomma M. Dervins-Ravault D. Newton H. J. (2010). Analysis of the *Legionella longbeachae* genome and transcriptome uncovers unique strategies to cause Legionnaires' disease. PLoS Genet. 6:e1000851 10.1371/journal.pgen.1000851 20174605 PMC2824747

[B15] Cazalet C. Rusniok C. Brüggemann H. Zidane N. Magnier A. Ma L. (2004). Evidence in the *Legionella pneumophila* genome for exploitation of host cell functions and high genome plasticity. Nat. Genet. 36, 1165–1173 10.1038/ng1447 15467720

[B16] Chatfield C. H. Cianciotto N. P. (2007). The secreted pyomelanin pigment of *Legionella pneumophila* confers ferric reductase activity. Infect. Immun. 75, 4062–4070 10.1128/IAI.00489-07 17548481 PMC1951983

[B17] Chen D. E. Podell S. Sauer J. D. Swanson M. S. Saier M. H. Jr. (2008). The phagosomal nutrient transporter (Pht) family. Microbiology 154, 42–53 10.1099/mic.0.2007/010611-0 18174124

[B18] Chien M. Morozova I. Shi S. Sheng H. Chen J. Gomez S. M. (2004). The genomic sequence of the accidental pathogen *Legionella pneumophila*. Science 305, 1966–1968 10.1126/science.1099776 15448271

[B19] Dalebroux Z. D. Edwards R. L. Swanson M. S. (2009). SpoT governs *Legionella pneumophila* differentiation in host macrophages. Mol. Microbiol. 71, 640–658 10.1111/j.1365-2958.2008.06555.x 19040633

[B20] Dalebroux Z. D. Yagi B. F. Sahr T. Buchrieser C. Swanson M. S. (2010). Distinct roles of ppGpp and DksA in *Legionella pneumophila* differentiation. Mol. Microbiol. 76, 200–219 10.1111/j.1365-2958.2010.07094.x 20199605 PMC2908999

[B21] D'Auria G. Jimenez-Hernandez N. Peris-Bondia F. Moya A. Latorre A. (2010). *Legionella pneumophila* pangenome reveals strain-specific virulence factors. BMC Genomics 11:181 10.1186/1471-2164-11-181 20236513 PMC2859405

[B22] Debroy S. Dao J. Soderberg M. Rossier O. Cianciotto N. P. (2006). *Legionella pneumophila* type II secretome reveals unique exoproteins and a chitinase that promotes bacterial persistence in the lung. Proc. Natl. Acad. Sci. U.S.A. 103, 19146–19151 10.1073/pnas.0608279103 17148602 PMC1748190

[B23] Edwards R. L. Dalebroux Z. D. Swanson M. S. (2009). *Legionella pneumophila* couples fatty acid flux to microbial differentiation and virulence. Mol. Microbiol. 71, 1190–1204 10.1111/j.1365-2958.2008.06593.x 19170883 PMC13200437

[B24] Eisenreich W. Dandekar T. Heesemann J. Goebel W. (2010). Carbon metabolism of intracellular bacterial pathogens and possible links to virulence. Nat. Rev. Microbiol. 8, 401–412 10.1038/nrmicro2351 20453875

[B25] Eisenreich W. Heesemann J. Rudel T. Goebel W. (2013). Metabolic host responses to infection by intracellular bacterial pathogens. Front. Cell. Infect. Microbiol. 3:24 10.3389/fcimb.2013.00024 23847769 PMC3705551

[B26] Ensminger A. W. Isberg R. R. (2010). E3 ubiquitin ligase activity and targeting of BAT3 by multiple *Legionella pneumophila* translocated substrates. Infect. Immun. 78, 3905–3919 10.1128/IAI.00344-10 20547746 PMC2937443

[B27] Ewann F. Hoffman P. S. (2006). Cysteine metabolism in *Legionella pneumophila*: characterization of an L-cystine-utilizing mutant. Appl. Environ. Microbiol. 72, 3993–4000 10.1128/AEM.00684-06 16751507 PMC1489648

[B28] Eylert E. Herrmann V. Jules M. Gillmaier N. Lautner M. Buchrieser C. (2010). Isotopologue profiling of *Legionella pneumophila*: role of serine and glucose as carbon substrates. J. Biol. Chem. 285, 22232–22243 10.1074/jbc.M110.128678 20442401 PMC2903384

[B29] Eylert E. Schar J. Mertins S. Stoll R. Bacher A. Goebel W. (2008). Carbon metabolism of *Listeria monocytogenes* growing inside macrophages. Mol. Microbiol. 69, 1008–1017 10.1111/j.1365-2958.2008.06337.x 18627458

[B30] Faucher S. P. Mueller C. A. Shuman H. A. (2011). *Legionella pneumophila* transcriptome during intracellular multiplication in human macrophages. Front. Microbiol. 2:60 10.3389/fmicb.2011.00060 21747786 PMC3128937

[B31] Feeley J. C. Gibson R. J. Gorman G. W. Langford N. C. Rasheed J. K. Mackel D. C. (1979). Charcoal-yeast extract agar: primary isolation medium for *Legionella pneumophila*. J. Clin. Microbiol. 10, 437–441 393713 10.1128/jcm.10.4.437-441.1979 PMC273193

[B32] Fields B. S. Benson R. F. Besser R. E. (2002). *Legionella* and Legionnaires' disease: 25 years of investigation. Clin. Microbiol. Rev. 15, 506–526 10.1128/CMR.15.3.506-526.2002 12097254 PMC118082

[B33] Finsel I. Ragaz C. Hoffmann C. Harrison C. F. Weber S. Van Rahden V. A. (2013). The *Legionella* effector RidL inhibits retrograde trafficking to promote intracellular replication. Cell Host Microbe 14, 38–50 10.1016/j.chom.2013.06.001 23870312

[B34] Fonseca M. V. Sauer J. D. Crepin S. Byrne B. Swanson M. S. (2014). The *phtC*-*phtD* locus equips *Legionella pneumophila* for thymidine salvage and replication in macrophages. Infect. Immun. 82, 720–730 10.1128/IAI.01043-13 24478086 PMC3911408

[B35] Gal-Mor O. Segal G. (2003a). Identification of CpxR as a positive regulator of *icm* and *dot* virulence genes of *Legionella pneumophila*. J. Bacteriol. 185, 4908–4919 10.1128/JB.185.16.4908-4919.2003 12897011 PMC166489

[B36] Gal-Mor O. Segal G. (2003b). The *Legionella pneumophila* GacA homolog (LetA) is involved in the regulation of *icm* virulence genes and is required for intracellular multiplication in *Acanthamoeba castellanii*. Microb. Pathog. 34, 187–194 10.1016/S0882-4010(03)00027-5 12668142

[B37] Gao L. Y. Harb O. S. Abu Kwaik Y. (1997). Utilization of similar mechanisms by *Legionella pneumophila* to parasitize two evolutionarily distant host cells, mammalian macrophages and protozoa. Infect. Immun. 65, 4738–4746 9353059 10.1128/iai.65.11.4738-4746.1997 PMC175680

[B38] Garcia M. T. Jones S. Pelaz C. Millar R. D. Abu Kwaik Y. (2007). *Acanthamoeba polyphaga* resuscitates viable non-culturable *Legionella pneumophila* after disinfection. Environ. Microbiol. 9, 1267–1277 10.1111/j.1462-2920.2007.01245.x 17472639

[B39] Garduno R. A. Garduno E. Hiltz M. Hoffman P. S. (2002). Intracellular growth of *Legionella pneumophila* gives rise to a differentiated form dissimilar to stationary-phase forms. Infect. Immun. 70, 6273–6283 10.1128/IAI.70.11.6273-6283.2002 12379706 PMC130304

[B40] Gebran S. J. Yamamoto Y. Newton C. Klein T. W. Friedman H. (1994). Inhibition of *Legionella pneumophila* growth by gamma interferon in permissive A/J mouse macrophages: role of reactive oxygen species, nitric oxide, tryptophan, and iron(III). Infect. Immun. 62, 3197–3205 8039889 10.1128/iai.62.8.3197-3205.1994 PMC302946

[B41] George J. R. Pine L. Reeves M. W. Harrell W. K. (1980). Amino acid requirements of *Legionella pneumophila*. J. Clin. Microbiol. 11, 286–291 6769947 10.1128/jcm.11.3.286-291.1980 PMC273381

[B42] Gillmaier N. Gotz A. Schulz A. Eisenreich W. Goebel W. (2012). Metabolic responses of primary and transformed cells to intracellular *Listeria monocytogenes*. PLoS ONE 7:e52378 10.1371/journal.pone.0052378 23285016 PMC3528701

[B43] Glöckner G. Albert-Weissenberger C. Weinmann E. Jacobi S. Schunder E. Steinert M. (2007). Identification and characterization of a new conjugation/type IVA secretion system (*trb*/*tra*) of *Legionella pneumophila* Corby localized on two mobile genomic islands. Int. J. Med. Microbiol. 298, 411–428 10.1016/j.ijmm.2007.07.012 17888731

[B44] Greub G. Raoult D. (2004). Microorganisms resistant to free-living amoebae. Clin. Microbiol. Rev. 17, 413–433 10.1128/CMR.17.2.413-433.2004 15084508 PMC387402

[B45] Hales L. M. Shuman H. A. (1999a). *Legionella pneumophila* contains a type II general secretion pathway required for growth in amoebae as well as for secretion of the Msp protease. Infect. Immun. 67, 3662–3666 10377156 10.1128/iai.67.7.3662-3666.1999 PMC116561

[B46] Hales L. M. Shuman H. A. (1999b). The *Legionella pneumophila rpoS* gene is required for growth within *Acanthamoeba castellanii*. J. Bacteriol. 181, 4879–4889 10438758 10.1128/jb.181.16.4879-4889.1999 PMC93975

[B47] Hammer B. K. Swanson M. S. (1999). Co-ordination of *Legionella pneumophila* virulence with entry into stationary phase by ppGpp. Mol. Microbiol. 33, 721–731 10.1046/j.1365-2958.1999.01519.x 10447882

[B48] Hammer B. K. Tateda E. S. Swanson M. S. (2002). A two-component regulator induces the transmission phenotype of stationary-phase *Legionella pneumophila*. Mol. Microbiol. 44, 107–118 10.1046/j.1365-2958.2002.02884.x 11967072 PMC13220096

[B49] Haneburger I. Hilbi H. (2013). Phosphoinositide lipids and the *Legionella* pathogen vacuole. Curr. Top. Microbiol. Immunol. 376, 155–173 10.1007/82_2013_341 23918172

[B50] Harada E. Iida K. Shiota S. Nakayama H. Yoshida S. (2010). Glucose metabolism in *Legionella pneumophila*: dependence on the Entner-Doudoroff pathway and connection with intracellular bacterial growth. J. Bacteriol. 192, 2892–2899 10.1128/JB.01535-09 20363943 PMC2876494

[B51] Hartel T. Eylert E. Schulz C. Petruschka L. Gierok P. Grubmüller S. (2012). Characterization of central carbon metabolism of *Streptococcus pneumoniae* by isotopologue profiling. J. Biol. Chem. 287, 4260–4274 10.1074/jbc.M111.304311 22167202 PMC3281726

[B52] Herrmann V. Eidner A. Rydzewski K. Blädel I. Jules M. Buchrieser C. (2010). GamA is a eukaryotic-like glucoamylase responsible for glycogen- and starch-degrading activity of *Legionella pneumophila*. Int. J. Med. Microbiol. 301, 133–139 10.1016/j.ijmm.2010.08.016 20965781

[B53] Hilbi H. Haas A. (2012). Secretive bacterial pathogens and the secretory pathway. Traffic 13, 1187–1197 10.1111/j.1600-0854.2012.01344.x 22340894

[B54] Hilbi H. Hoffmann C. Harrison C. F. (2011). *Legionella* spp. outdoors: colonization, communication and persistence. Environ. Microbiol. Rep. 3, 286–296 10.1111/j.1758-2229.2011.00247.x 23761274

[B55] Hoffmann C. Finsel I. Otto A. Pfaffinger G. Rothmeier E. Hecker M. (2014a). Functional analysis of novel Rab GTPases identified in the proteome of purified *Legionella*-containing vacuoles from macrophages. Cell. Microbiol. 16, 1034–1052 10.1111/cmi.12256 24373249

[B56] Hoffmann C. Harrison C. F. Hilbi H. (2014b). The natural alternative: protozoa as cellular models for *Legionella* infection. Cell. Microbiol. 16, 15–26 10.1111/cmi.12235 24168696

[B57] Hovel-Miner G. Faucher S. P. Charpentier X. Shuman H. A. (2010). ArgR-regulated genes are derepressed in the *Legionella*-containing vacuole. J. Bacteriol. 192, 4504–4516 10.1128/JB.00465-10 20622069 PMC2937375

[B58] Hovel-Miner G. Pampou S. Faucher S. P. Clarke M. Morozova I. Morozov P. (2009). SigmaS controls multiple pathways associated with intracellular multiplication of *Legionella pneumophila*. J. Bacteriol. 191, 2461–2473 10.1128/JB.01578-08 19218380 PMC2668410

[B59] Hubber A. Roy C. R. (2010). Modulation of host cell function by *Legionella pneumophila* type IV effectors. Annu. Rev. Cell. Dev. Biol. 26, 261–283 10.1146/annurev-cellbio-100109-104034 20929312

[B60] Isberg R. R. O'Connor T. J. Heidtman M. (2009). The *Legionella pneumophila* replication vacuole: making a cosy niche inside host cells. Nat. Rev. Microbiol. 7, 13–24 10.1038/nrmicro1967 19011659 PMC2631402

[B61] Ivanov S. S. Charron G. Hang H. C. Roy C. R. (2010). Lipidation by the host prenyltransferase machinery facilitates membrane localization of *Legionella pneumophila* effector proteins. J. Biol. Chem. 285, 34686–34698 10.1074/jbc.M110.170746 20813839 PMC2966084

[B62] James B. W. Mauchline W. S. Dennis P. J. Keevil C. W. Wait R. (1999). Poly-3-hydroxybutyrate in *Legionella pneumophila*, an energy source for survival in low-nutrient environments. Appl. Environ. Microbiol. 65, 822–827 9925622 10.1128/aem.65.2.822-827.1999 PMC91101

[B63] James B. W. Mauchline W. S. Fitzgeorge R. B. Dennis P. J. Keevil C. W. (1995). Influence of iron-limited continuous culture on physiology and virulence of *Legionella pneumophila*. Infect. Immun. 63, 4224–4230 7591051 10.1128/iai.63.11.4224-4230.1995 PMC173600

[B64] Johnson W. Varner L. Poch M. (1991). Acquisition of iron by *Legionella pneumophila*: role of iron reductase. Infect. Immun. 59, 2376–2381 1904841 10.1128/iai.59.7.2376-2381.1991 PMC258021

[B65] Joseph B. Mertins S. Stoll R. Schar J. Umesha K. R. Luo Q. (2008). Glycerol metabolism and PrfA activity in *Listeria monocytogenes*. J. Bacteriol. 190, 5412–5430 10.1128/JB.00259-08 18502850 PMC2493282

[B66] Kessler A. Schell U. Sahr T. Tiaden A. Harrison C. Buchrieser C. (2013). The *Legionella pneumophila* orphan sensor kinase LqsT regulates competence and pathogen-host interactions as a component of the LAI-1 circuit. Environ. Microbiol. 15, 646–662 10.1111/j.1462-2920.2012.02889.x 23033905

[B67] Khosravi-Darani K. Mokhtari Z. B. Amai T. Tanaka K. (2013). Microbial production of poly(hydroxybutyrate) from C(1) carbon sources. Appl. Microbiol. Biotechnol. 97, 1407–1424 10.1007/s00253-012-4649-0 23306640

[B68] Koubar M. Rodier M. H. Frère J. (2013). Involvement of minerals in adherence of *Legionella pneumophila* to surfaces. Curr. Microbiol. 66, 437–442 10.1007/s00284-012-0295-0 23292133

[B69] Kozak N. A. Buss M. Lucas C. E. Frace M. Govil D. Travis T. (2010). Virulence factors encoded by *Legionella longbeachae* identified on the basis of the genome sequence analysis of clinical isolate D-4968. J. Bacteriol. 192, 1030–1044 10.1128/JB.01272-09 20008069 PMC2812971

[B70] Lau H. Y. Ashbolt N. J. (2009). The role of biofilms and protozoa in *Legionella pathogenesis*: implications for drinking water. J. Appl. Microbiol. 107, 368–378 10.1111/j.1365-2672.2009.04208.x 19302312

[B71] Liles M. R. Edelstein P. H. Cianciotto N. P. (1999). The prepilin peptidase is required for protein secretion by and the virulence of the intracellular pathogen *Legionella pneumophila*. Mol. Microbiol. 31, 959–970 10.1046/j.1365-2958.1999.01239.x 10048038

[B72] Liles M. R. Scheel T. A. Cianciotto N. P. (2000). Discovery of a nonclassical siderophore, legiobactin, produced by strains of *Legionella pneumophila*. J. Bacteriol. 182, 749–757 10.1128/JB.182.3.749-757.2000 10633110 PMC94339

[B73] Lomma M. Dervins-Ravault D. Rolando M. Nora T. Newton H. J. Sansom F. M. (2010). The *Legionella pneumophila* F-box protein Lpp2082 (AnkB) modulates ubiquitination of the host protein parvin B and promotes intracellular replication. Cell. Microbiol. 12, 1272–1291 10.1111/j.1462-5822.2010.01467.x 20345489

[B74] Lynch D. Fieser N. Gloggler K. Forsbach-Birk V. Marre R. (2003). The response regulator LetA regulates the stationary-phase stress response in *Legionella pneumophila* and is required for efficient infection of *Acanthamoeba castellanii*. FEMS Microbiol. Lett. 219, 241–248 10.1016/S0378-1097(03)00050-8 12620627

[B75] Mengaud J. M. Horwitz M. A. (1993). The major iron-containing protein of *Legionella pneumophila* is an aconitase homologous with the human iron-responsive element-binding protein. J. Bacteriol. 175, 5666–5676 8366052 10.1128/jb.175.17.5666-5676.1993 PMC206625

[B76] Molofsky A. B. Swanson M. S. (2003). *Legionella pneumophila* CsrA is a pivotal repressor of transmission traits and activator of replication. Mol. Microbiol. 50, 445–461 10.1046/j.1365-2958.2003.03706.x 14617170 PMC13227487

[B77] Molofsky A. B. Swanson M. S. (2004). Differentiate to thrive: lessons from the *Legionella pneumophila* life cycle. Mol. Microbiol. 53, 29–40 10.1111/j.1365-2958.2004.04129.x 15225301 PMC13218203

[B78] Nasrallah G. K. Riveroll A. L. Chong A. Murray L. E. Lewis P. J. Garduno R. A. (2011). *Legionella pneumophila* requires polyamines for optimal intracellular growth. J. Bacteriol. 193, 4346–4360 10.1128/JB.01506-10 21742865 PMC3165514

[B79] Pearce M. M. Cianciotto N. P. (2009). *Legionella pneumophila* secretes an endoglucanase that belongs to the family-5 of glycosyl hydrolases and is dependent upon type II secretion. FEMS Microbiol. Lett. 300, 256–264 10.1111/j.1574-6968.2009.01801.x 19817866 PMC2766432

[B80] Pine L. George J. R. Reeves M. W. Harrell W. K. (1979). Development of a chemically defined liquid medium for growth of *Legionella pneumophila*. J. Clin. Microbiol. 9, 615–626 39086 10.1128/jcm.9.5.615-626.1979 PMC275359

[B81] Poch M. T. Johnson W. (1993). Ferric reductases of *Legionella pneumophila*. Biometals 6, 107–114 10.1007/BF00140111 8358204

[B82] Price C. T. Al-Khodor S. Al-Quadan T. Abu Kwaik Y. (2010a). Indispensable role for the eukaryotic-like ankyrin domains of the ankyrin B effector of *Legionella pneumophila* within macrophages and amoebae. Infect. Immun. 78, 2079–2088 10.1128/IAI.01450-09 20194593 PMC2863551

[B83] Price C. T. Al-Khodor S. Al-Quadan T. Santic M. Habyarimana F. Kalia A. (2009). Molecular mimicry by an F-box effector of *Legionella pneumophila* hijacks a conserved polyubiquitination machinery within macrophages and protozoa. PLoS Pathog. 5:e1000704 10.1371/journal.ppat.1000704 20041211 PMC2790608

[B84] Price C. T. Al-Quadan T. Santic M. Jones S. C. Abu Kwaik Y. (2010b). Exploitation of conserved eukaryotic host cell farnesylation machinery by an F-box effector of *Legionella pneumophila*. J. Exp. Med. 207, 1713–1726 10.1084/jem.20100771 20660614 PMC2916131

[B85] Price C. T. Al-Quadan T. Santic M. Rosenshine I. Abu Kwaik Y. (2011). Host proteasomal degradation generates amino acids essential for intracellular bacterial growth. Science 334, 1553–1557 10.1126/science.1212868 22096100

[B86] Rasis M. Segal G. (2009). The LetA-RsmYZ-CsrA regulatory cascade, together with RpoS and PmrA, post-transcriptionally regulates stationary phase activation of *Legionella pneumophila* Icm/Dot effectors. Mol. Microbiol. 72, 995–1010 10.1111/j.1365-2958.2009.06705.x 19400807

[B87] Ratledge C. Dover L. G. (2000). Iron metabolism in pathogenic bacteria. Annu. Rev. Microbiol. 54, 881–941 10.1146/annurev.micro.54.1.881 11018148

[B88] Reeves M. W. Pine L. Hutner S. H. George J. R. Harrell W. K. (1981). Metal requirements of *Legionella pneumophila*. J. Clin. Microbiol. 13, 688–695 6785311 10.1128/jcm.13.4.688-695.1981 PMC273860

[B89] Ristroph J. D. Hedlund K. W. Gowda S. (1981). Chemically defined medium for *Legionella pneumophila* growth. J. Clin. Microbiol. 13, 115–119 7462408 10.1128/jcm.13.1.115-119.1981 PMC273733

[B90] Robey M. Cianciotto N. P. (2002). *Legionella pneumophila feoAB* promotes ferrous iron uptake and intracellular infection. Infect. Immun. 70, 5659–5669 10.1128/IAI.70.10.5659-5669.2002 12228295 PMC128349

[B91] Rohmer L. Hocquet D. Miller S. I. (2011). Are pathogenic bacteria just looking for food? Metabolism and microbial pathogenesis. Trends. Microbiol. 19, 341–348 10.1016/j.tim.2011.04.003 21600774 PMC3130110

[B92] Rothmeier E. Pfaffinger G. Hoffmann C. Harrison C. F. Grabmayr H. Repnik U. (2013). Activation of Ran GTPase by a *Legionella* effector promotes microtubule polymerization, pathogen vacuole motility and infection. PLoS Pathog. 9:e1003598 10.1371/journal.ppat.1003598 24068924 PMC3777869

[B93] Rowbotham T. J. (1986). Current views on the relationships between amoebae, legionellae and man. Isr. J. Med. Sci. 22, 678–689 3793451

[B94] Sahr T. Brüggemann H. Jules M. Lomma M. Albert-Weissenberger C. Cazalet C. (2009). Two small ncRNAs jointly govern virulence and transmission in *Legionella pneumophila*. Mol. Microbiol. 72, 741–762 10.1111/j.1365-2958.2009.06677.x 19400772 PMC2888818

[B95] Sauer J. D. Bachman M. A. Swanson M. S. (2005). The phagosomal transporter A couples threonine acquisition to differentiation and replication of *Legionella pneumophila* in macrophages. Proc. Natl. Acad. Sci. U.S.A. 102, 9924–9929 10.1073/pnas.0502767102 15998735 PMC1174991

[B96] Schell U. Kessler A. Hilbi H. (2014). Phosphorylation signalling through the *Legionella* quorum sensing histidine kinases LqsS and LqsT converges on the response regulator LqsR. Mol. Microbiol. 92, 1039–1055 10.1111/mmi.12612 24720786

[B97] Schroeder G. N. Petty N. K. Mousnier A. Harding C. R. Vogrin A. J. Wee B. (2010). *Legionella pneumophila* strain 130b possesses a unique combination of type IV secretion systems and novel Dot/Icm secretion system effector proteins. J. Bacteriol. 192, 6001–6016 10.1128/JB.00778-10 20833813 PMC2976443

[B98] Schunder E. Gillmaier N. Kutzner E. Herrmann V. Lautner M. Heuner K. (2014). Amino acid uptake and metabolism of *Legionella pneumophila* hosted by *Acanthamoeba castellanii*. J. Biol. Chem. 289, 21040–21054 10.1074/jbc.M114.570085 24904060 PMC4110309

[B99] Spirig T. Tiaden A. Kiefer P. Buchrieser C. Vorholt J. A. Hilbi H. (2008). The *Legionella* autoinducer synthase LqsA produces an a-hydroxyketone signaling molecule. J. Biol. Chem. 283, 18113–18123 10.1074/jbc.M801929200 18411263 PMC2440625

[B100] Starkenburg S. R. Casey J. M. Cianciotto N. P. (2004). Siderophore activity among members of the *Legionella* genus. Curr. Microbiol. 49, 203–207 10.1007/s00284-004-4342-3 15386105

[B101] Steeb B. Claudi B. Burton N. A. Tienz P. Schmidt A. Farhan H. (2013). Parallel exploitation of diverse host nutrients enhances *Salmonella* virulence. PLoS Pathog. 9:e1003301 10.1371/journal.ppat.1003301 23633950 PMC3636032

[B102] Steinert M. Emody L. Amann R. Hacker J. (1997). Resuscitation of viable but nonculturable *Legionella pneumophila* Philadelphia JR32 by *Acanthamoeba castellanii*. Appl. Environ. Microbiol. 63, 2047–2053 9143134 10.1128/aem.63.5.2047-2053.1997 PMC168494

[B103] Steinert M. Flügel M. Schuppler M. Helbig J. H. Supriyono A. Proksch P. (2001). The Lly protein is essential for p-hydroxyphenylpyruvate dioxygenase activity in *Legionella pneumophila*. FEMS Microbiol. Lett. 203, 41–47 10.1111/j.1574-6968.2001.tb10818.x 11557138

[B104] Tesh M. J. Miller R. D. (1981). Amino acid requirements for *Legionella pneumophila* growth. J. Clin. Microbiol. 13, 865–869 6113250 10.1128/jcm.13.5.865-869.1981 PMC273905

[B105] Tesh M. J. Miller R. D. (1983). Arginine biosynthesis in *Legionella pneumophila*: absence of N-acetylglutamate synthetase. Can. J. Microbiol. 29, 1230–1233 10.1139/m83-190 6652583

[B106] Tesh M. J. Morse S. A. Miller R. D. (1983). Intermediary metabolism in *Legionella pneumophila*: utilization of amino acids and other compounds as energy sources. J. Bacteriol. 154, 1104–1109 6133845 10.1128/jb.154.3.1104-1109.1983 PMC217580

[B107] Tiaden A. Hilbi H. (2012). a-Hydroxyketone synthesis and sensing by *Legionella* and *Vibrio*. Sensors 12, 2899–2919 10.3390/s120302899 22736983 PMC3376566

[B108] Tiaden A. Spirig T. Carranza P. Brüggemann H. Riedel K. Eberl L. (2008). Synergistic contribution of the *Legionella pneumophila lqs* genes to pathogen-host interactions. J. Bacteriol. 190, 7532–7547 10.1128/JB.01002-08 18805977 PMC2576672

[B109] Tiaden A. Spirig T. Hilbi H. (2010). Bacterial gene regulation by a-hydroxyketone signaling. Trends. Microbiol. 18, 288–297 10.1016/j.tim.2010.03.004 20382022

[B110] Tiaden A. Spirig T. Weber S. S. Brüggemann H. Bosshard R. Buchrieser C. (2007). The *Legionella pneumophila* response regulator LqsR promotes host cell interactions as an element of the virulence regulatory network controlled by RpoS and LetA. Cell. Microbiol. 9, 2903–2920 10.1111/j.1462-5822.2007.01005.x 17614967

[B111] Urwyler S. Brombacher E. Hilbi H. (2009a). Endosomal and secretory markers of the *Legionella*-containing vacuole. Commun. Integr. Biol. 2, 107–109 Available online at: http://www.landesbioscience.com/journals/cib/article/7713 19704903 10.4161/cib.7713 PMC2686358

[B112] Urwyler S. Nyfeler Y. Ragaz C. Lee H. Mueller L. N. Aebersold R. (2009b). Proteome analysis of *Legionella* vacuoles purified by magnetic immunoseparation reveals secretory and endosomal GTPases. Traffic 10, 76–87 10.1111/j.1600-0854.2008.00851.x 18980612

[B113] Vikram H. R. Bia F. J. (2002). Severe *Legionella pneumophila* pneumonia in a patient with iron overload. Scand. J. Infect. Dis. 34, 772–774 10.1080/00365540260348608 12477334

[B114] Viswanathan V. K. Edelstein P. H. Pope C. D. Cianciotto N. P. (2000). The *Legionella pneumophila iraAB* locus is required for iron assimilation, intracellular infection, and virulence. Infect. Immun. 68, 1069–1079 10.1128/IAI.68.3.1069-1079.2000 10678909 PMC97250

[B115] Weiss E. Peacock M. Williams J. (1980). Glucose and glutamate metabolism of *Legionella pneumophila*. Curr. Microbiol. 4, 1–6 10.1007/BF02602882

[B116] Wieland H. Ullrich S. Lang F. Neumeister B. (2005). Intracellular multiplication of *Legionella pneumophila* depends on host cell amino acid transporter SLC1A5. Mol. Microbiol. 55, 1528–1537 10.1111/j.1365-2958.2005.04490.x 15720558

[B117] Williams R. J. (2012). Iron in evolution. FEBS Lett. 586, 479–484 10.1016/j.febslet.2011.05.068 21704034

[B118] Zamboni N. Fendt S. M. Ruhl M. Sauer U. (2009). (13)C-based metabolic flux analysis. Nat. Protoc. 4, 878–892 10.1038/nprot.2009.58 19478804

[B119] Zheng H. Chatfield C. H. Liles M. R. Cianciotto N. P. (2013). Secreted pyomelanin of *Legionella pneumophila* promotes bacterial iron uptake and growth under iron-limiting conditions. Infect. Immun. 81, 4182–4191 10.1128/IAI.00858-13 23980114 PMC3811826

[B120] Zusman T. Aloni G. Halperin E. Kotzer H. Degtyar E. Feldman M. (2007). The response regulator PmrA is a major regulator of the Icm/Dot type IV secretion system in *Legionella pneumophila* and *Coxiella burnetii*. Mol. Microbiol. 63, 1508–1523 10.1111/j.1365-2958.2007.05604.x 17302824

[B121] Zusman T. Gal-Mor O. Segal G. (2002). Characterization of a *Legionella pneumophila relA* insertion mutant and toles of RelA and RpoS in virulence gene expression. J. Bacteriol. 184, 67–75 10.1128/JB.184.1.67-75.2002 11741845 PMC134777

